# β-carotene and *Bacillus thuringiensis* insecticidal protein differentially modulate feeding behaviour, mortality and physiology of European corn borer (*Ostrinia nubilalis*)

**DOI:** 10.1371/journal.pone.0246696

**Published:** 2021-02-16

**Authors:** Patricia Sarai Girón-Calva, Carmen Lopez, Alfonso Albacete, Ramon Albajes, Paul Christou, Matilde Eizaguirre

**Affiliations:** 1 Department of Plant Production and Forestry Sciences, University of Lleida-Agrotecnio Center, Lleida, Spain; 2 Instituto Murciano de Investigación y Desarrollo Agrario y Alimentario, Murcia, Spain; 3 ICREA, Catalan Institute for Research and Advanced Studies, Barcelona, Spain; Nigde Omer Halisdemir University, TURKEY

## Abstract

Maize with enhanced β-carotene production was engineered to counteract pervasive vitamin A deficiency in developing countries. Second-generation biofortified crops are being developed with additional traits that confer pest resistance. These include crops that can produce *Bacillus thuringiensis* Berliner (Bt) insecticidal proteins. Currently, it is unknown whether β-carotene can confer fitness benefits through to insect pests, specifically through altering *Ostrinia nubilalis* foraging behaviour or development in the presence of Bt insecticidal toxin. Therefore the effects of dietary β-carotene plus Bt insecticidal protein on feeding behaviour, mortality, and physiology in early and late instars of *O*. *nubilalis* larvae were investigated. The results of two-choice experiments showed that irrespective of β-carotene presence, at day five 68%-90% of neonates and 69%-77% of fifth-instar larvae avoided diets with Cry1A protein. Over 65% of neonate larvae preferred to feed on diets with β-carotene alone compared to 39% of fifth-instar larvae. Higher mortality (65%-97%) in neonates fed diets supplemented with β-carotene alone and in combination with Bt protein was found, whereas <36% mortality was observed when fed diets without supplemented β-carotene or Bt protein. Diets with both β-carotene and Bt protein extended 25 days the larval developmental duration from neonate to fifth instar (compared to Bt diets) but did not impair larval or pupal weight. Juvenile hormone and 20-hydroxyecdysone regulate insect development and their levels were at least 3-fold higher in larvae fed diets with β-carotene for 3 days. Overall, these results suggest that the effects of β-carotene and Bt protein on *O*. *nubilalis* is dependent on larval developmental stage. This study is one of the first that provides insight on how the interaction of novel traits may modulate crop susceptibility to insect pests. This understanding will in turn inform the development of crop protection strategies with greater efficacy.

## Introduction

Plant chemical composition, including primary and secondary metabolites, mediates plant-insect interactions. Herbivorous insects selectively feed on plants that provide necessary nutrients to fuel growth, development, and reproduction [1]. Gustatory neurons housed within sensilla on mouthparts (e.g. mandibles, maxillae, and labial palps) aid insects in distinguishing between nutrients and non-nutrients in plants [2]. Thus, stimulating or deterrent plant compounds influence insect feeding choices [3]. However, plant chemical composition is context dependent. Biotic and abiotic factors drive spatio-temporal variation in plant chemistry [4] that then affects insect performance and development [5].

Crops containing Cry1A insecticidal protein reduces negative agricultural effects of lepidopteran insect pests [6]. However, some insects have larvae that wander before settling on a plant part from which to feed. These insects could potentially evade toxins through their movement. An example is the European corn borer *Ostrinia nubilalis* (Hübner), whose neonate caterpillars can avoid feeding on Bt plants [7, 8].

The agriculture industry is developing biofortified crops that produce nutritionally important molecules, such as vitamins or antioxidants [9, 10]. However, little information is available concerning how these novel traits affect plant-herbivore interactions [11]. One engineered strain of maize known as HC produces extraordinarily high β-carotene concentrations (up to 60 μg/g dry weight in endosperm) [9, 11]. In insects, carotenoids and their derivatives provide body coloration [12], are precursors to visual pigment chromophores [13] and act as antioxidants [14, 15]. However, insects generally acquire carotenoids from their diets [16]. Some herbivorous insects may selectively feed on plants enriched in antioxidants or other nutrients to bolster their immunity against environmental stress [15]. These include early-stage *Grammia incorrupta* (Hy. Edwards, 1881) larvae, which selectively feed on *Malva parviflora* L. when parasitized, thus gaining fitness benefits and an enhanced immune response from the plant’s high antioxidant levels [15]. Examples such as this raise the question of whether β-carotene production in novel crops could influence herbivore insect fitness and have the unintended effect of attracting insect pests.

An improved version of HC maize (HC × Bt) was engineered with the *B*. *thuringiensis* (Bt) Cry1A protein to confer insect-pest resistance [11, 17]. Bt insecticidal protein consumption increases oxidative stress in larvae midgut [18]. However, insects possess enzymatic and non-enzymatic defence systems that counteract oxidative stress [19]. Additionally, insects may utilise dietary antioxidants to counteract Bt protein toxicity. For example, the nitric oxide scavenger and phenoloxidase inhibitor glutathione delayed mortality in gypsy moth (*Lymantria dispar* L.) larvae fed Bt Cry1A toxin alone (MVPII; 20 μg), although overall mortality was not significantly affected [20]. Furthermore, *O*. *nubilalis* larvae fed HC × Bt diets had lower mortality than larvae fed diets of plants containing only Cry1A [17]. When added to Bt diets, β-carotene reduced susceptibility of fifth-instar larvae to Cry1A through scavenging reactive oxygen species (ROS) produced during Cry1A detoxification [17]. Other antioxidants show a similar antagonistic behaviour to Cry1A. Quercetin, incorporated into artificial diets together with Cry1A protein, reduced mortality of *Helicoverpa armigera* (Hübner) neonate and fifth-instar larvae [21]. The insect endocrine system is also involved in activating oxidative stress responses [22]. For instance, hormonal regulation of oxidative stress correlates with extended developmental periods of larvae that were fed different xenobiotics [23]. Juvenile hormone II (JH) and 20-hydroxyecdysone (20HE) interplay to control insect growth, differentiation, metamorphosis and reproduction [22]. JH and 20HE content in insects show a dynamic pattern. JH is secreted by the corpora allata and its content typically increases sharply three days after moulting followed by a rapid decrease [24, 25]. Pulses of 20HE triggers growth cessation or pupation [26, 27]. Cry1A protein appear to impact the insect endocrine system, as shown by a decreased JH titre in *H*. *armigera* fed diets containing Cry1A protein [24]. However, little is known on the interaction of β-carotene and Cry1A insecticidal protein in affecting food choices and the endocrine system of insect pests, and whether these novel traits influence early- and late-instar larvae equally.

This study investigated whether β-carotene and Cry1A insecticidal protein interact to alter the behaviour, performance, and physiology of *O*. *nubilalis* larvae, a major pest of maize and target for Cry1A. In a series of experiments, the hypotheses that larvae prefer diets with β-carotene independently of Cry1A presence and that β-carotene improves fitness of larvae challenged with Cry1A in a dose-dependent manner were tested. To do so, mortality, larval/pupal weight, and duration of development from neonate to fifth instar were measured. Lastly, it was investigated the endocrinological mechanism of β-carotene and Cry1A influence on insect development. Specifically, 20HE and JH were measured. The results of this study should provide insights on how novel traits interact with each other to alter crop susceptibility to insect pests. Better understanding of these interactions will inform genetic engineering projects aimed at developing crops with greater sustainability and durability.

## Materials and methods

### Insect rearing

Diapausing *O*. *nubilalis* larvae (Lepidoptera: Crambidae) were collected after maize harvest, with permission of the owner (Josep Pique), during November 2018 in a commercial fields near Lleida (41°37′00″N 00°38′00″E) with no Bt maize cultivation. Larvae were placed inside plastic containers and kept in a room maintained at 25°C under a 16:8 h light:dark photoperiod to terminate diapause. A 1 × 1 × 1 cm^3^ cube of semiartificial diet previously described by Eizaguirre and Albajes [28] was kept inside each container for ad libitum feeding and to keep humidity high (>60%). Upon reaching the pupal stage, sex identification was carried out by observing the morphological traits of pupae under a microscope: in males the genital opening is located after the fourth posterior abdominal segment whereas in females the genital opening is located after the third posterior abdominal segment. Male and female pupae were kept into individual containers with high humidity (>60%). After adults emerged, females and males were placed together into a cage for mating and oviposition. Third-to-fifth generation laboratory-reared neonates and fifth-instar (L5) larvae were used in this study.

### Plant material

Freeze-dried leaves of commercial Bt maize DKC6667Y (Cry1A) MON810 were added to artificial diets, thus incorporating Cry1A insecticidal protein. Non-Bt maize DKC6666 (isogenic) was the control. Leaves from Bt and non-Bt maize were previously collected in fields near Lleida. Leaves were cut into small strips and main veins removed, then freeze-dried in a vacuum drier (Gamma 2–16 LSC plus, CHRIST, Osterode am Harz, Germany) and ground to fine powder in a Thermomix®. The powder was stored at −80°C until use.

Semiartificial diets used for insect rearing were modified to include Cry1A and different β-carotene concentrations (denoted in diets as β) (Table 1). The proportion of freeze-dried leaf material to β-carotene aimed to mimic concentrations found in existing maize varieties [17]. Cry1A concentrations in Bt and Bt-β diets were verified with the Agdia Bt-Cry1Ab/Cry1Ac kit (Agdia Inc., Elkhart, Indiana, US). Cry1A standards at 100, 75, 50, 25, 15, 8, and 2 ng/mL were used for calibration. Measurements were made with an Infinite F Nano^+^ spectrophotometer (TECAN, Männedorf, Switzerland) at 650 nm.

**Table 1:** Composition of diets used in this study.

Components	% composition per 100 g of diet			
	No-Bt	No-Bt-β	Bt	Bt-β
Corn flour^a^	10.8	10.8	10.8	10.8
Freeze-dried leaves	0.2^h^	0.2^h^	0.2^i^	0.2^i^
Wheat germ^b^	3	3	3	3
Nutritional yeast^c^	3	3	3	3
Ascorbic acid^d^	0.5	0.5	0.5	0.5
Sorbic acid^e^	0.2	0.2	0.2	0.2
Agar-Agar^f^	1.6	1.6	1.6	1.6
Water	80.7	80.7	80.7	80.7
β-carotene^g^	-	60 or 6 mg	-	60 or 6 mg

^a,b,c^ Sorribas (Polinyá, Spain)

^d,e^ Panreac (Castellar del Vallès, Spain)

^f^ ROKO (Llanera, Spain)

^g^ Sigma-Aldrich, Inc. (Darmstadt, Germany)

^h^ No Bt maize

^i^ Bt maize

### Experiment 1: Larval food choices

Diet-preference assays were performed to test whether β-carotene and Cry1A (hereafter Bt), alone or in combination, affect *O*. *nubilalis* food choices. It was also tested whether food preferences diverge between early (neonate) and late (L5) larval instars. First, diets with leaves of either non-Bt or Bt maize to neonate and L5 larvae (non-Bt *vs*. Bt diets) were offered. Then larval food preferences when β-carotene was added (non-Bt *vs*. non-Bt-β and Bt *vs*. Bt-β) were evaluated. Lastly, the properties of Bt insecticidal protein and β-carotene as attractants or deterrents (non-Bt-β *vs*. Bt and non-Bt *vs*. Bt-β) were compared. Neonate larvae were tested in a small, lidded plastic container (5 cm diameter × 3 cm height). In it, 0.5 × 0.5 × 0.5 cm^3^ samples of two different diets were arranged equidistantly. Fifth-instar larvae were tested in a Petri dish (15 cm diameter × 2 cm height) with two 1 × 1 × 1 cm^3^ samples of different diets equidistant from each other. Only one larva was used per container/Petri dish. Individuals were released into the middle of the test arena before it was sealed (Petri dishes with Parafilm). Larval activity was recorded at four different evaluation intervals (6 h, 1, 2, and 5 d after release into the arenas). Larval positioning was recorded: those on a diet cube were noted as selecting that diet, while those that did not approach either cube were noted as ‘no choice’. Sixty neonate larvae and 60 L5 larvae (n = 60) were used per combination.

### Experiment 2: Larval mortality, weight, and development duration

No-choice feeding assays were conducted to determine whether β-carotene can alter the effect of Bt insecticidal protein on first-instar larvae. L5 larvae had been tested in a previous experiment [17].

For the first trial, the influence of Bt and non-Bt diets with and without 60 mg of β-carotene per 100 g of diet (see Table 1 for other components) on larval survival, weight, and developmental duration was determined. Neonate larvae were placed individually into plastic lidded containers (5 cm diameter × 3 cm height) along with a diet cube (non-Bt, Bt, non-Bt-β, or Bt-β). Sixty larvae were used per diet (n = 60). Old diets were replaced periodically with fresh ones. Larvae were inspected every 2 d and mortality recorded. When larvae moulted to L5, we recorded weight, and the duration in days between L1 and early L5. Neonate larvae had higher mortality rate when fed diets with β-carotene. In a second trial, a dose-dependent effect of β-carotene was tested by comparing the effects of 6 mg β-carotene per 100 g diet with 60 mg. Feeding assays were conducted as in the first trial, with 30 neonate larvae used per diet (n = 30). Larval mortality was recorded every 2 d. Pupal weight and development duration in days from neonate to pupa were also measured.

### Experiment 3: Effect of β-carotene and Bt insecticidal protein on hormonal balance of late-instar larvae

#### Larval feeding

To determine the influence of β-carotene, alone or with Bt insecticidal protein, on *O*. *nubilalis* physiology, the variation in JH and 20HE was measured, both important to insect growth and development. L5 larvae reared individually in small plastic containers (5 cm diameter × 3 cm height) and fed since L1 on artificial diets without β-carotene or Bt were used in this experiment. On L5-day 0 (when larvae moulted to fifth instar), we fed them non-Bt and Bt diets with or without 6 mg β-carotene. Two groups of 30 larvae were given one diet type. After feeding for 1 d (L5-day 1), one group of 30 larvae was frozen in liquid nitrogen. Weight was recorded and frozen larvae were randomly distributed into six groups with five larvae each (n = 6). The remaining 30 larvae were subjected to the same treatment after 3 d of feeding (L5-day 3) (n = 6). Frozen larvae were kept at −80°C until use.

#### Extraction of JH and 20HE

Six replicate samples per tested diet for both L5-day 1 and L5-day 3. First, five frozen larvae were placed into a universal extraction bag with synthetic intermediate layer (12 × 15 cm; BIOREBA, Reinach, Switzerland). Next, 2 mL of 100% methanol was added into each bag, and contents were mixed with a hand homogeniser (BIOREBA, Reinach, Switzerland). Finally, separate 0.5 mL aliquots of homogenised larvae were collected and placed into individual 2 mL Eppendorf tubes® for JH and 20HE extraction.

Juvenile hormone was extracted following published procedures [29] with modifications. Briefly, 0.5 mL of isooctane on ice was added to Eppendorf tubes® containing 0.5 mL of larvae/methanol. The mixture was kept at 24°C for 5 min. After centrifugation at 10,000 rpm for 1 min, the supernatant was collected, and 30 ng of methoprene was added as an internal standard. Next, 100 μL of 2% NaCl solution and 300 μL of hexane were added to the mixture before vortexing and resting for 5 min at 24°C. After a second centrifugation at 3200 rpm for 5 min, the hexane-isooctane phase was transferred into an amber glass vial. This extraction with hexane was repeated three times to obtain 900 μL of hexane. The hexane-isooctane mixture was vacuum-dried in a centrifugal concentrator (Model SPD 131 DDA-230, Fisher Scientific, Madrid, Spain).

The steroid hormone 20HE was extracted following procedures described in Honda et al. [30] with modifications. After centrifugation at 10000 rpm and 4°C for 10 min, methanol was collected into a new Eppendorf tube and the pellet was used for a second extraction with methanol. This methanol mixture was vacuum-dried under reduced pressure. Chloroform/water (1:2 v/v, 300 μL) was added to the dried pellet, and after centrifugation at 10,000 rpm and 4°C for 5 min, the aqueous phase was collected into an amber glass vial. The extraction with chloroform/water was repeated twice. The aqueous phase was then dried under reduced pressure. Dry pellets from JH and 20HE extracts were dissolved in 1 mL of methanol/water (20/80, v/v) solution using an ultrasonic bath (JP Selecta S.A., Barcelona, Spain). Dissolved samples were passed through 13 mm diameter Millex filters (pore size: 0.22 μm pore) (Millipore, Bedford, MA, USA).

#### Analysis of JH and 20HE using UPLC-MS

Filtrated extract (10 μL) was injected in an Accela Series UHPLC (ThermoFisher Scientific, Waltham, MA, USA) coupled to an Exactive mass spectrometer (Thermo FisherScientific, Waltham, MA, USA). A chromatographic C18 Accucore column (2.1 × 100 mm, 1.5 μm, ThermoFisher Scientific, Waltham, MA, USA) was used. Flow rate was set to 300 μL/min. Run time was 10 min, with the elution gradient starting at 20% MeOH (0–1 min), linearly increasing to 80% MeOH (1–6 min), remaining at 80% MeOH (6–8 min), then decreasing and staying at 20% MeOH for re-equilibration. Mass detection was performed in negative mode at a scan range 90–500 m/z. A heated electrospray ionisation source was used with spray voltage of 4 kV and capillary temperature of 275°C. Mass spectra were obtained using Xcalibur version 2.2 (ThermoFisher Scientific, Waltham, MA, USA). For JH and 20HE quantification, calibration curves were constructed with 1× weighted linear fitting for each component (0.01, 0.1, 1, and 10 μg l^-1^). Recovery percentages (92–95%) were calculated from the internal standard methoprene.

### Statistical analyses

Larvae that did not make a choice in experiment 1 were excluded from the analysis. Proportion of time spent on each diet cube for each larva were calculated in Microsoft Excel 2016. Significant deviation from expected distribution was determined with Pearson Chi-square tests.

In experiment 2, the mortality was calculated as percentage for each trial but statistical analysis to compare diets (β-carotene variation) was performed with a binomial approximation using the total number of dead and live larvae in the calculations. Data were pooled if significant differences were absent. Analysis of variance (ANOVA) was used to test for the effects of β-carotene (β), Bt, and their interaction on larval development time and larval weight. Differences between diets containing β-carotene were assessed with Student’s *t*-test.

In experiment 3, differences in hormone titres were determined using three- and two-way ANOVAS, followed by Tukey’s post-hoc test for multiple comparisons; Bt, β-carotene, and day were main factors. Student’s *t*-tests were used to assess differences between days. Kruskal-Wallis *H* tests, with diet as main factor, was used to assess non-parametric data when applicable. Pairwise comparisons were performed with Tukey post hoc tests and Bonferroni corrections. Mann-Whitney *U* test was used to assess differences in weight gain between days.

To determine whether parametric or non-parametric tests were suitable, normality and homogeneity of variance assumptions were tested with Shapiro-Wilk and Levene’s tests. All statistical tests were performed in JMP ® Pro version 14.1.0.

## Results

### Experiment 1. Larval food choices

The results of ELISA confirmed that diets with Bt-maize leaves had the target Bt concentration of 31.5 ± 6 (mean ± SE) μg/g fresh weight, which is similar to the concentration of Bt protein found in leaves and kernels of commercial Bt maize DKC6667Y and HC × Bt maize [17].

**Fig 1:**
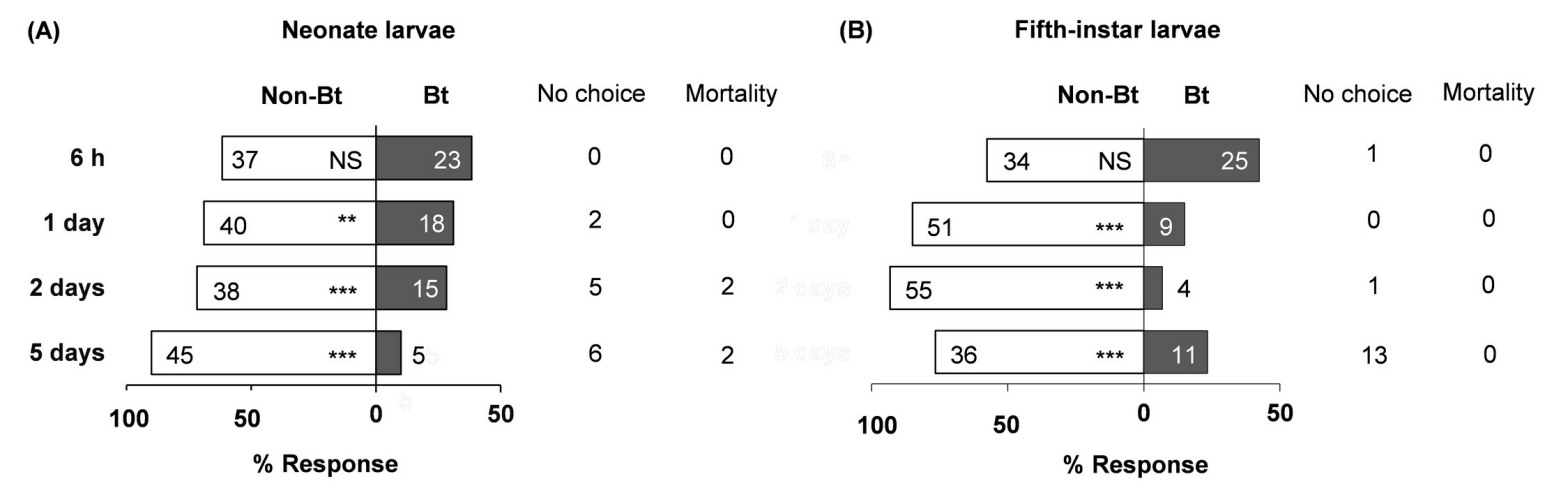
Responsiveness of *Ostrinia nubilalis* larvae to Bt insecticidal protein. Food preferences (in percentages) of neonate (A) and fifth-instar (B) larvae for diets with Cry1A protein (Bt) or without (Non-Bt). Sixty individuals from each instar were tested (n = 60), and their location (on or off diet) was recorded at four time points (6 h, 1, 2, and 5 d from start of experiment). Number of larvae per diet is depicted within each bar. Off-diet or dead larvae were excluded from the analysis and their counts shown next to each bar under ‘No choice’ and ‘Mortality’, respectively. Asterisks indicate significant differences between diets (*X*^*2*^, *P = 0.05, **P = 0.01, ***P < 0.001). NS, not significant. Detailed information on diet components is in the Materials and Methods section.

**Fig 2:**
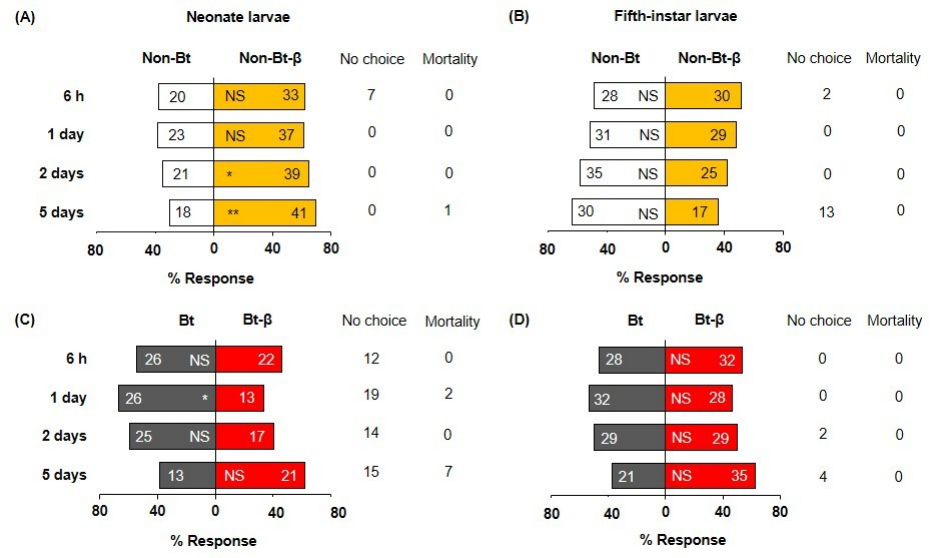
Food choices of *Ostrinia nubilalis* larvae when β-carotene is incorporated into Bt and non-Bt diets. β-carotene (denoted as β) was added to semi-artificial diets with Bt protein or without (Non-Bt), then offered to neonate (A, C) and fifth-instar (B, D) larvae. Sixty individuals per instar were tested (n = 60), and their location (on or off diet) were recorded at four time points (6 h, 1, 2, and 5 d). Each bar displays number of larvae on each diet. Off-diet or dead larvae were excluded from the analysis; their counts are shown next to each bar under ‘No choice’ and ‘Mortality’, respectively. Asterisks indicate significant differences between diets (*X*^*2*^, *P = 0.05, **P = 0.01, ***P < 0.001). NS, not significant. See the Materials and Methods section for detailed diet components.

**Fig 3:**
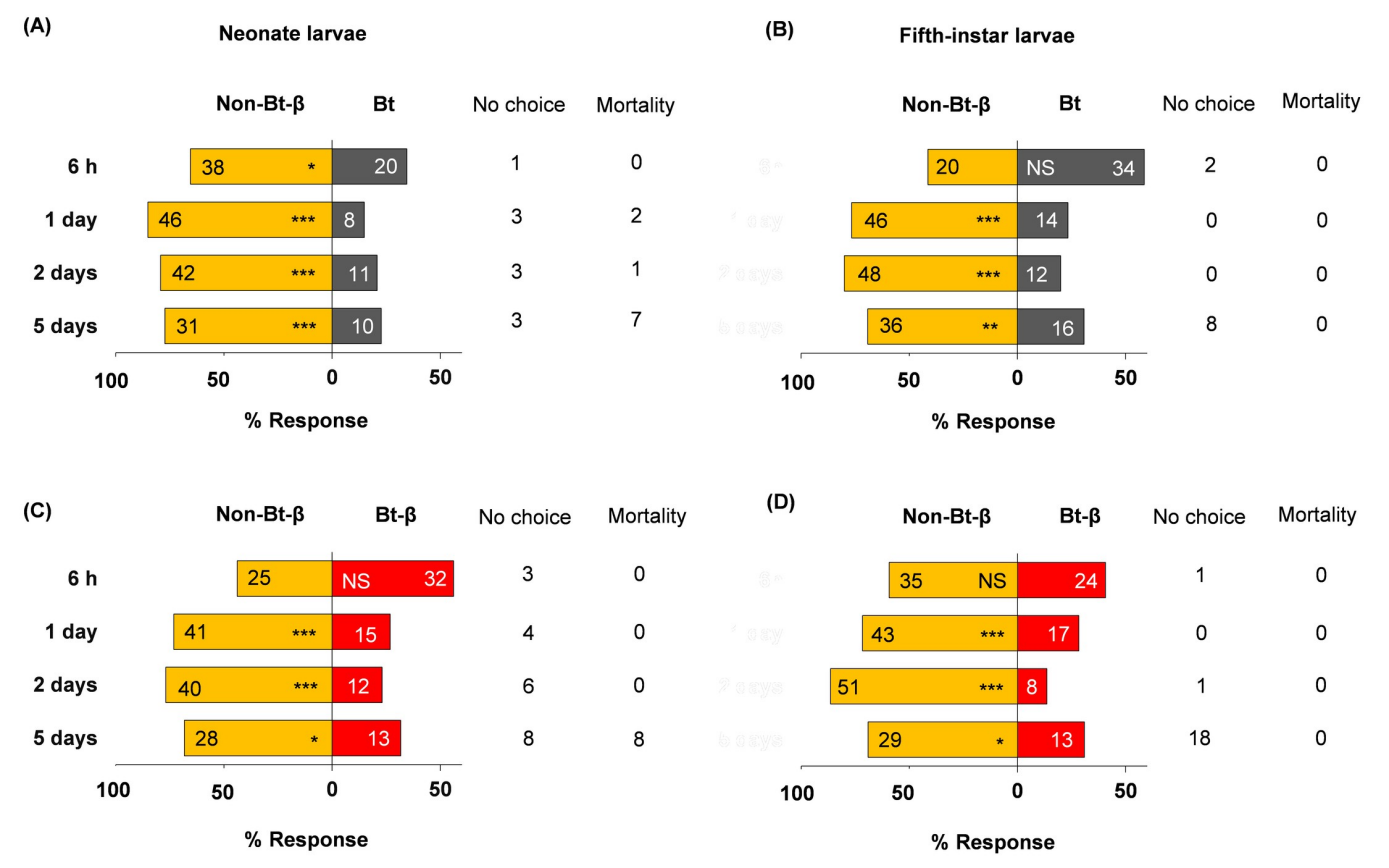
Food preferences of *Ostrinia nubilalis*. Food choices (in percentages) of neonate (A, C) and fifth-instar (B, D) larvae when β-carotene (β) is incorporated into semi-artificial diets with Cry1A protein (Bt) or without (Non-Bt). Sixty individuals of each instar were tested (n = 60), and their location (on or off diet) were recorded over time (6 h, 1, 2 and 5 d). Each bar displays number of larvae per diet. Off-diet or dead larvae were excluded from the analysis; their counts are shown next to each bar under ‘No choice’ and ‘Mortality’, respectively. Asterisks indicate significant differences between diets (*X*^*2*^, *P = 0.05, **P = 0.01, ***P < 0.001). NS, not significant. Diet composition under Materials and Methods.

Neonate larvae showed no preference between Bt and non-Bt diets 6 h after exposure began, but significantly preferred non-Bt at days 1, 2, and 5 (Fig 1A, S1 Table). Fifth-instar larvae showed a similar trend (Fig 1B). At day 5, 13 L5 larvae were off diet cubes and ready to moult; they were excluded from further analyses. Adding β-carotene to non-Bt and Bt diets altered neonate choice only in the absence of Bt protein. Neonates significantly preferred to feed on non-Bt-β diet over non-Bt diet at days 2 and 5 (Fig 2A, S1 Table), but had no significant preference between Bt and Bt-β diets (Fig 2C, S1 Table). Fifth-instar larvae exhibited no preference between Bt and Bt-β diets at any time point (Fig 2B and 2D, S1 Table). The feeding-deterrent properties of Bt we observed that neonates and L5 larvae largely preferred non-Bt-β diets over Bt diets, irrespective of β-carotene presence were further corroborated (Fig 3A–3D, S1 Table). Collectively, these assays demonstrate that Bt protein deters *O*. *nubilalis* larvae independently of developmental stage or β-carotene presence. However, β-carotene alone appears to stimulate feeding in early instars, but not late ones.

### Experiment 2. Larval mortality, weight, and development time

**Fig 4:**
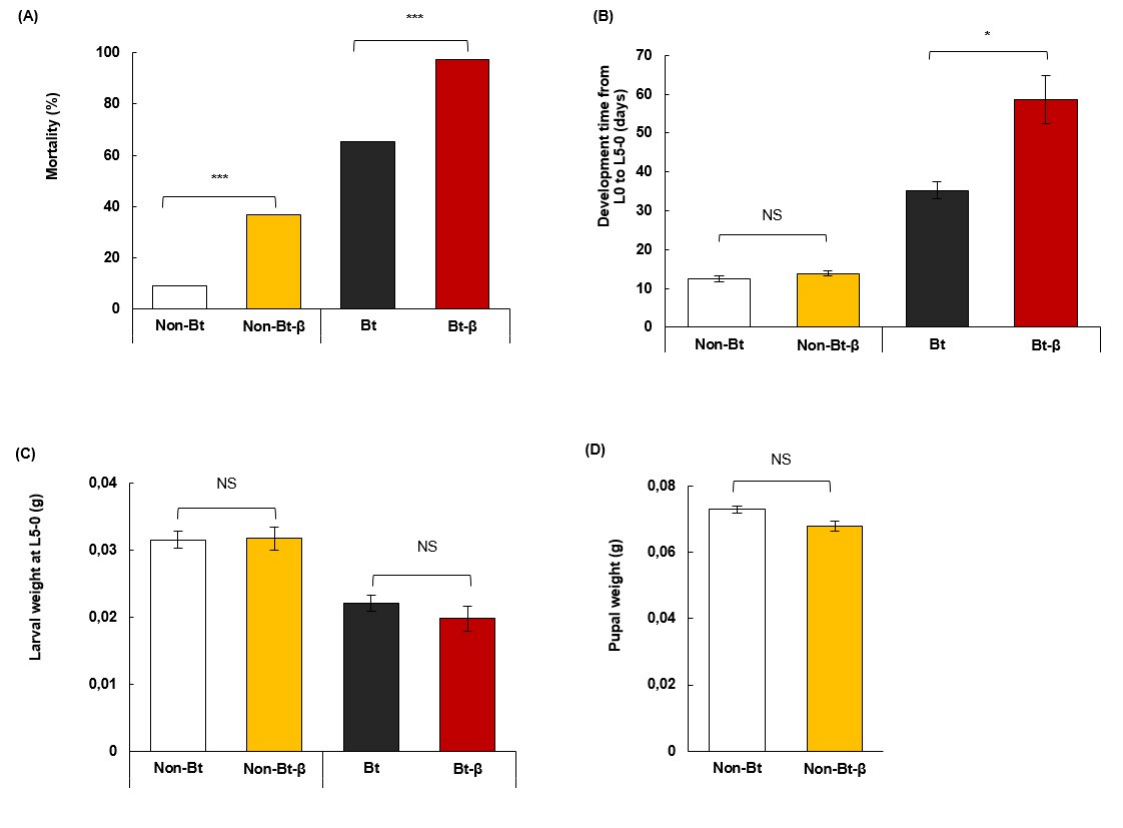
Performance of *O*. *nubilalis* larvae fed β-carotene and Bt insecticidal protein. Neonate larvae were given diets with Bt protein (Bt) or without (Non-Bt) and supplemented with or without β-carotene (β). (A) Mortality after 3 weeks of feeding. (B) Developmental period from neonate (L0) to fifth instar (L5). (C) Larval weight. (D) Pupal weight. Larvae were kept individually in plastic containers and fed one diet type. Asterisks indicate significant differences between diets with or without β (*P = 0.05, **P = 0.01, ***P < 0.001). Error bars correspond to standard error (±SE).

We pooled data for 60 and 6 mg of β-carotene/100 g of diet because we observed no significant differences in their effects on larvae (S2 Table). Feeding on the Bt diet, with or without β-carotene, extended larval development and decreased larval weight (Fig 4). Adding β-carotene to the non-Bt diet increased larval mortality by four times, but did not extend larval developmental duration, nor did it increase weight in L5 larvae or pupae (Fig 4A–4D, S3–S5 Tables). Larval mortality increased to 97% when β-carotene was added to the Bt diet, which caused 60% mortality (Fig 4A, S3 Table). β-Carotene in a Bt diet also prolonged larval development 25 days (compared to Bt diet) but did not change larval weight (Fig 4B and 4C, S4 and S5 Tables). At the end of the experiment, >60% of larvae fed non-Bt and non-Bt-β diets pupated, whereas none on Bt or Bt-β diets pupated.

### Experiment 3. Effect of β-carotene and Bt insecticidal protein on hormonal balance of late-instar larvae

A three-way ANOVA found significant effects of hormone measurement timing (i.e. 1 or 3 d after larvae began to feed on experimental diets) (S6 Table).

**Fig 5:**
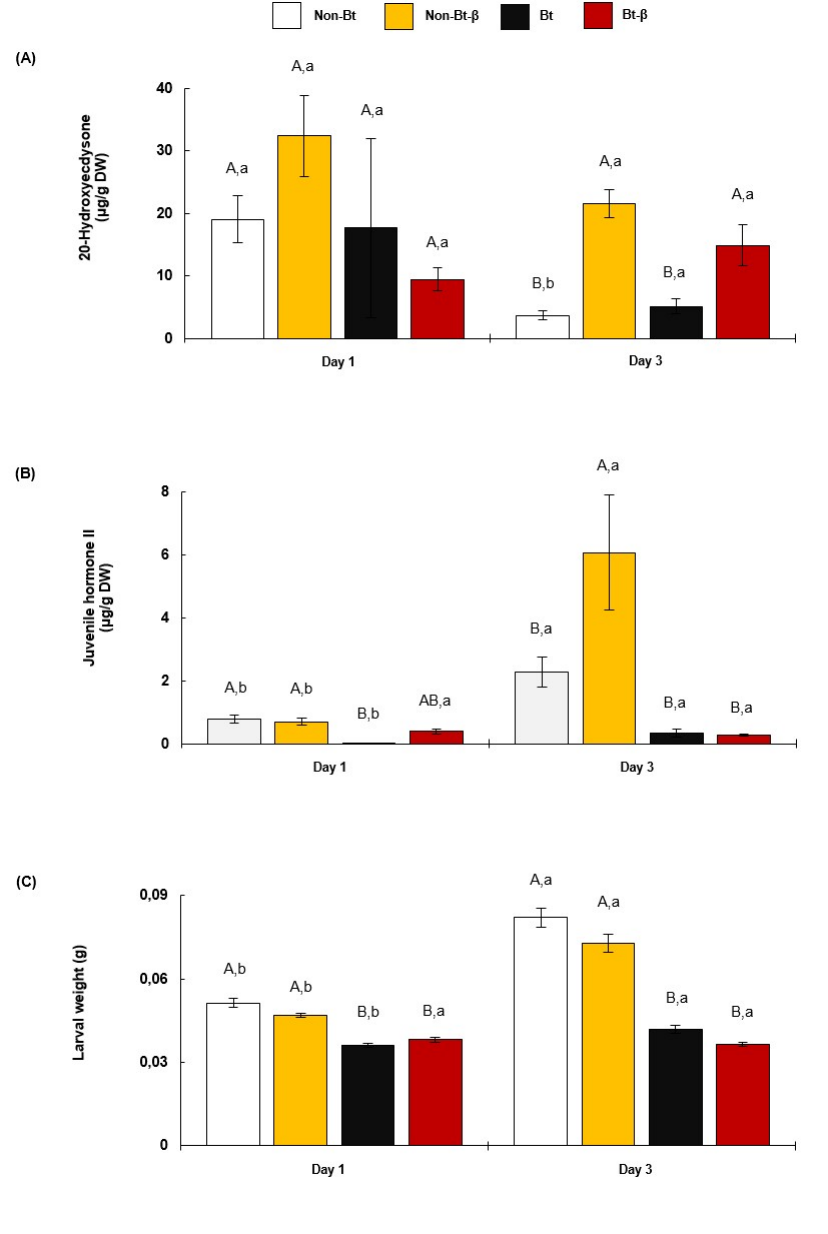
20-Hydroxyecdysone (20HE) and Juvenile Hormone (JH) concentrations (mean ± SE) in *O*. *nubilalis* fifth-instar larvae fed β-carotene and Bt insecticidal protein. Hormones 20HE (A) and JH (B) were quantified in larvae fed diets with Bt protein (Bt) or without (Non-Bt) and with or without 6 mg of β-carotene (β) per 100 g of diet, for 1–3 d. Thirty larvae were weighed at day 1. For hormone extraction, six groups of five insects were formed (n = 6). A different set of larvae were similarly treated at day 3. Different capital letters above bars indicate significant differences between diets within each day. Different lowercase letters indicate significant differences within diets between days. DW, dry weight.

No significant differences in 20HE titre were found between larvae fed non-Bt, non-Bt-β, Bt, or Bt-β diets for 1 d (Fig 5A, S7 Table). However, larvae fed for 3 d on non-Bt-β and Bt-β had higher 20HE titre than larvae fed on non-Bt and Bt (Fig 5A, S7 Table). Student’s *t*-tests revealed that 20HE titre was similar across larvae on non-Bt-β and Bt-β diets at days 1 and 3, whereas 20HE decreased significantly in larvae on non-Bt and Bt diets at day 3 (Fig 5A, S8 Table).

Larvae fed non-Bt and non-Bt-β diets for 1 d had significantly higher JH titre than those fed Bt and Bt-β diets (Fig 5B, S7 Table). After 3 d, JH titre remained lower in larvae fed Bt diets than in larvae fed non-Bt diets, independently of β-carotene content (Fig 5B, S7 Table). Student’s *t*-tests revealed increased JH titre among larvae fed non-Bt, non-Bt-β, and Bt for 3 d compared with 1 d (Fig 5B, S8 Table). Larvae Bt-β did not differ in JH titre after 1 or 3 d (Fig 5B, S8 Table).

Irrespective of β-carotene presence, larvae fed non-Bt and non-Bt-β diets for 1 d gained significantly more weight than larvae fed Bt-containing diets (Fig 5C, S9 Table). Larvae gained weight being fed for 3 d on non-Bt, non-Bt-β, and Bt diets, no weight gain between day 1 and day 3 was observed in larvae fed Bt-β (Fig 5C, S10 Table).

## Discussion

This study was aimed to investigate whether β-carotene and Cry1A insecticidal protein interact to alter the feeding behaviour, mortality, and hormonal balance of *O*. *nubilalis* larvae, a major pest of maize expressing Cry1A protein. *O*. *nubilalis* larvae was found to avoid diets that incorporated Bt insecticidal toxin, independently of larval stage within 24 h, corroborating Goldstein et al. [7], but there was not neonates or L5 larvae moving off from non-Bt diets after 24 h, indicating that they accepted the food source [31]. However, other authors have observed that early-instar larvae are slower to avoid toxins than late instars [32].

Beside their importance in insect nutrition [33], carotenoids also function as visual cues for foraging herbivorous insects to evaluate plant quality. Silencing the phytoene desaturase gene, encoding a key enzyme in plant carotenoid synthesis, affected the behaviour and fitness of herbivorous insects [34]. However, most studies examining β-carotene cannot address whether the compound is a feeding stimulant for herbivorous insects, because the experiments are no-choice tests. The dual-choice tests in this study found that neonate larvae did not discriminate between non-Bt and non-Bt-β during the first 48 h of exposure. Subsequently, neonates clearly preferred non-Bt diets with β-carotene. In contrast, L5 larvae fed indiscriminately on diets with and without β-carotene throughout the experiment. Although feeding behaviour of *O*. *nubilalis* larvae is different in early instar (external feeder) from that late instars (stem boring) [8], no evidence was found that β-carotene irritated early or late instar larvae because the insects did not move off these diets [31].

However, neonates and late-instar larvae are assumed to use the same mechanisms when locating hosts [35]. Their food selection depends on sensitivity to specific chemical stimuli from host plants, including deterrent or phagostimulatory secondary metabolites [2]. Further research can elucidate whether gustatory sensilla responses differ between early and late instars of lepidopteran larvae, as well as whether β-carotene stimulates *O*. *nubilalis* larval sensilla. Early-instar larvae may be more sensitive to specific phagostimulants than late-instar larvae as several authors suggest for *Heliothis virescens* [36, 37].

In most of the cases, larval food selection is the result of combined stimuli [38]. The fact that neonates and L5 larvae actively avoided Bt-containing diets independently of β-carotene suggests that the latter compound does not mask Bt presence; thus, its phagostimulatory properties for neonates are context-dependent. Food-mixing behaviour of herbivorous insects in nature appears to involve both nutrient balancing and toxin dilution [39]. However, food mixing was not observed in larvae with access to Bt and Bt-β diets. Instead, more neonates stopped eating within 6 h, while L5 larvae remained on their selected diet. Although previous research suggested that β-carotene contributes to Bt detoxification in *O*. *nubilalis* larvae [17], there was no evidence that larvae feeding on Bt-β diets were less irritated by Bt protein. In the previous work performed by Zanga et al. [17], larvae were fed in only one type of diet, without possibility of moving off the diet so favouring the insect detoxification mechanisms. Similarly, in this study, larvae feeding on Bt or Bt- β diets moved to non-Bt-β diets after 24 h and did not return to Bt diets. Although these results cannot be extrapolated to novel Bt+carotenoid-expressing crops, these results still suggests that β-carotene does not compromise the deterrent properties of Bt insecticidal toxin nor reduce its effectiveness against *O*. *nubilalis*.

β-Carotene increased the mortality of early-instar larvae independently of Bt content, and this effect was not dose-dependent. Carotenoids and other dietary antioxidants improve insect development [33, 40] or survival [41]. Carotenoids also enhance insect immune response to biotic stress, such as parasitism [14, 42]. In a previous study with *O*. *nubilalis* larvae [17], β-carotene-supplemented diets enhanced immune response to Bt insecticidal protein in a non-dose-dependent manner, with mortality decreasing by 20–35%. On the contrary, in the present study a detrimental effect of β-carotene was detected as the non-Bt diet with β-carotene increased larval mortality, while β-carotene and Bt appear to increase mortality even further through a synergistic interaction. This discrepancy is probably attributable to different instar used in both studies and to differences in the feeding history. In this study, larvae were reared since emergence on experimental diets (with and without β-carotene or Bt toxin), whereas Zanga et al. [17] tested L5 larvae reared previously, from neonate to L5, on a control artificial diet. However, present results are in agreement with those of Eichenseer et al. [43] with *Helicoverpa zea* or Clark and Lampert [42] with *Trichoplusia ni*. These authors [42] suggested that growth rate differences between early and late instars are explainable via a trade-off between resource allocation to growth and to β-carotene modifications in early-instar larvae. Therefore, insect physiological factors and nutritional state may influence absorption and carotenoid accumulation [43–45].

The fact that in this study, β-carotene did not influence larval and pupal weight suggests that by the fifth larval stage, the insects had acquired enough resources to allocate towards β-carotene transformation without compromising growth. Therefore, β-carotene absorption and transformation likely impose additional fitness costs in early-instar larvae, leading to increased mortality so corroborating the differences found with the Zanga et al [17] study. Early-instar *O*. *nubilalis larvae* are also more susceptible to Bt insecticidal protein than older larvae, even at lower doses [46]. The susceptibility of first instars to Bt and additional fitness costs from β-carotene absorption and transformation might increase mortality in larvae fed Bt-β diets relative to larvae fed Bt-only diets.

Larvae fed Bt-β diets have a longer developmental period which may indicate that β-carotene contribute to Bt detoxification. This finding is consistent with the work of Zanga et al. [17], where *O*. *nubilalis* exhibited extended development when fed diets containing maize expressing both Bt protein and carotenoids. Overall increase in lifespan is associated with oxidative-stress onset and subsequent activation of enzymatic and non-enzymatic detoxification [20, 23].

Juvenile hormone and 20HE play pivotal roles in insect development. The former prevents metamorphosis through suppressing prothoracicotropic hormone in the brain, thus inhibiting the ecdysone synthesis and secretion in the haemolymph [47]. After achieving critical weight through JH esterase activity, JH is eliminated from the body [48]. During the final instar, a small 20HE pulse triggers growth cessation and marks entry into the wandering stage [26]. At approximately 5 d after the wandering stage begins, a much larger ecdysone pulse triggers pupation [27].

Feeding duration affected larval hormonal response to β-carotene of the *O*. *nubilalis* larvae. After 3 d feeding on the of the β-carotene diet, JH and 20HE content increased regardless of Bt presence or absence. Larval weight was unaffected by β-carotene presence. These higher 20HE and JH levels suggest that β-carotene affects regulation of their synthesis or degradation. Multiple processes can influence this regulation. For instance, in insects, activation of anti-oxidative stress responses is hormonally regulated [22]. Bt-induced oxidative stress lengthened development and lowered weight in *O*. *nubilalis* larvae but may also indicate increased antioxidant enzymatic activity [49]. Both JH and 20HE temporally regulate antioxidant enzyme expression. Exposure to increasing 20HE levels elevated glutathione *S*-transferase (GST) mRNA *in Bombyx mori* [50]. The increase of JH and 20HE due to β-carotene ingestion probably directly influences ROS scavenging during Bt detoxification. A combination of elevated hormone levels and antioxidant capacity might have boosted anti-oxidative responses in larvae fed Bt and β-carotene. As a result, larvae did not achieve the critical weight for pupation, and larval duration increased to allow Bt mitigation by the antioxidant system.

Recently, Enya et al. [51] suggested that glutathione, the main substrate of GSTs, has a primary role in ecdysteroid synthesis in *D*. *melanogaster*, although the precise mechanism is unknown. Glutathione, like β-carotene, is an important antioxidant that contributes to maintaining redox homeostasis and mitigating oxidative stress. Therefore, it can be speculated that dietary β-carotene may interact with other insect antioxidants to reduce oxidative stress stemming and Bt protein detoxification so favouring 20HE synthesis. However, a more complete hormone profile of *O*. *nubilalis* late instars would be required to determine β-carotene’s exact effects.

Overall, the results of this study suggest that larval instar alters the influence of β-carotene on *O*. *nubilalis*. Neonate larvae do not have all the metabolic resources for carotenoid absorption, transport, and transformation so, feeding on β-carotene leads to increased mortality. In contrast, late-instar larvae gain fitness benefits from β-carotene because they probably possess more resources for absorption and transformation without compromising growth. Dietary β-carotene likely contributed significantly in reducing detoxification-related oxidative stress, changing JH and 20HE levels in the process. But the mechanisms behind this particular response to β-carotene is not fully understood.

This study is one of the few that provides insight on how insect pests may be affected by novel traits in biofortified crops, either alone or in combination. It should be note that biofortified commercial crops are likely to contain more than one carotenoid in different proportions. These combinations may interact uniquely with Bt insecticidal protein, exerting different effects on crop susceptibility to insect pests. Nevertheless, this study should benefit the development of successful crop protection programmes.

## Supporting information

S1 Table Pearson chi-square tests used to determine whether the distribution of larvae on each diet differ significantly from what was expected. (DOCX)

S2 Table Dose dependent effect of β-carotene on the mortality of early instar larvae of *Ostrinia nubilalis* fed with non-Br or Bt diets. (DOCX)

S3 Table Effect of β-carotene incorporated in non-Bt and Bt diets on the mortality of early instar larvae. Results of the approximation to the binomial test.
(DOCX)

S4 Table Two-way ANOVA of the combinative effect of Bt insecticidal protein and β-carotene (β) on the development of early instar larvae. (DOCX)

S5 Table Student’s t-tests on the effect of β-carotene in non-Bt and Bt diets on the performance of early instar larvae. (DOCX)

S6 Table Three-way ANOVA on the effects of day of quantification and the addition of Bt insecticidal toxin and β-carotene into diets on *O*. *nubilalis* hormone titre. (DOCX)

S7 Table Two-way ANOVA on the effects of Bt insecticidal toxin and β-carotene on *O*. *nubilalis* hormone titre within days of quantification. (DOCX)

S8 Table Student’s t-tests on the effect of β-carotene in non-Bt and Bt diets on the hormone titre of fifth instar larvae between days of quantification. (DOCX)

S9 Table Kruskal-Wallis *H*-test of the effect of diet on fifht instar larval weight at after 1 and 3 days of feeding. (DOCX)

S10 Table Mann-Whitney *U*-tests of the effect of diet on larval weight compared between days 1 and 3. (DOCX)

S1 File Data set needed to draw Figs 4 and 5. (XLSX)
