# Supplementary material for: β-carotene and Bacillus thuringiensis insecticidal protein differentially modulate feeding behaviour, mortality and physiology of European corn borer (Ostrinia nubilalis)
Source: PLoS One. 2021 Feb 16;16(2):e0246696. doi: 10.1371/journal.pone.0246696 (PMC7886157; doi:10.1371/journal.pone.0246696)
Supplement: S1 Table — (DOCX) [file pone.0246696.s001.docx]

| **S1 Table**. Pearson chi-square tests used to determine whether the distribution of larvae on each diet differ significantly from what was expected (*n* = 60) | | | | | | | | | | | |
| --- | --- | --- | --- | --- | --- | --- | --- | --- | --- | --- | --- |
|  |  | |  |  | |  |  | |  |  | |
|  | 6h | |  | 1 day | |  | 2 days | |  | 5 days | |
| Diets used in dual-choice tests | *X^2^* | *P* |  | *X^2^* | *P* |  | *X^2^* | *P* |  | *X^2^* | *P* |
| *Neonates* | | | | | | | | | | | |
| Non-Bt vs. Bt | 3.26 | 0.071 |  | 8.34 | 0.004 |  | 9.98 | 0.002 |  | 32 | < 0.001 |
| Non-Bt vs. Non-Bt-β | 3.18 | 0.074 |  | 3.26 | 0.071 |  | 5.4 | 0.02 |  | 8.9 | 0.003 |
| Bt vs. Bt-β | 0.33 | 0.56 |  | 4.33 | 0.037 |  | 1.52 | 0.217 |  | 1.88 | 0.17 |
| Non-Bt-β vs. Bt | 5.58 | 0.018 |  | 26.7 | < 0.001 |  | 18.3 | < 0.001 |  | 11.75 | 0.001 |
| Non-Bt-β vs. Bt-β | 0.86 | 0.356 |  | 12.07 | 0.001 |  | 15.07 | < 0.001 |  | 5.48 | 0.019 |
| *Fifth instar* | | | | | | | | | | | |
| Non-Bt vs. Bt | 1.37 | 0.241 |  | 29.4 | < 0.001 |  | 44.08 | < 0.001 |  | 11 | 0.001 |
| Non-Bt vs. Non-Bt-β | 0.276 | 0.599 |  | 0 | 1 |  | 1.66 | 0.197 |  | 3.59 | 0.06 |
| Bt vs. Bt-β | 0.267 | 0.606 |  | 0.267 | 0.606 |  | 0 | 1 |  | 2.48 | 0.115 |
| Non-Bt-β vs. Bt | 1.72 | 0.189 |  | 17.06 | < 0.001 |  | 21.6 | < 0.001 |  | 7.69 | 0.006 |
| Non-Bt-β vs. Bt-β | 2.051 | 0.152 |  | 11.26 | 0.001 |  | 31.3 | < 0.001 |  | 6.095 | 0.014 |
