# Supplementary material for: β-carotene and Bacillus thuringiensis insecticidal protein differentially modulate feeding behaviour, mortality and physiology of European corn borer (Ostrinia nubilalis)
Source: PLoS One. 2021 Feb 16;16(2):e0246696. doi: 10.1371/journal.pone.0246696 (PMC7886157; doi:10.1371/journal.pone.0246696)
Supplement: S2 Table — (DOCX) [file pone.0246696.s002.docx]

| **S2 Table.** Dose dependent effect of β-carotene on the mortality of early instar larvae of *Ostrinia nubilalis* fed with Non-Br or Bt diets^a^ (*n* = 30). The number of dead individuals is displayed on the table | | | | | |
| --- | --- | --- | --- | --- | --- |
|  | β-carotene content in 100 g of diet | | |  |  |
| Diet | 0 mg | 6 mg | 60 mg | *Z* | *P*^b^ |
| Non-Bt | 5 | 13 | 14 | 0.2595 | 0.79 |
| Bt | 28 | 27 | 26 |  |  |

^a^ For detailed information on diet composition, please see the Materials and Methods section in the main text.

^b^ Approximation to the binomial test, comparisons between diets with 6 and 60 mg of β-carotene.
