# Supplementary material for: β-carotene and Bacillus thuringiensis insecticidal protein differentially modulate feeding behaviour, mortality and physiology of European corn borer (Ostrinia nubilalis)
Source: PLoS One. 2021 Feb 16;16(2):e0246696. doi: 10.1371/journal.pone.0246696 (PMC7886157; doi:10.1371/journal.pone.0246696)
Supplement: S3 Table — Results of the approximation to the binomial test. (DOCX) [file pone.0246696.s003.docx]

| **S3 Table**. Effect of β-carotene incorporated in Non-Bt and Bt diets on the mortality of early instar larvae. Results of the approximation to the binomial test. | | |
| --- | --- | --- |
| Diet comparisons | *Z* | *P* |
| Non-Bt vs. Non-Bt-β | -4.45 | < 0.001 |
| Bt vs. Bt-β | -5.72 | < 0.001 |
