# Supplementary material for: β-carotene and Bacillus thuringiensis insecticidal protein differentially modulate feeding behaviour, mortality and physiology of European corn borer (Ostrinia nubilalis)
Source: PLoS One. 2021 Feb 16;16(2):e0246696. doi: 10.1371/journal.pone.0246696 (PMC7886157; doi:10.1371/journal.pone.0246696)
Supplement: S4 Table — (DOCX) [file pone.0246696.s004.docx]

| **S4 Table.** Two-way ANOVA of the combinative effect of Bt insecticidal protein and β-carotene (β) on the development of early instar larvae | | | | | | | |
| --- | --- | --- | --- | --- | --- | --- | --- |
|  | Larval development time | | |  | Larval weight | | |
| Variable | d.f | *F* | *P* |  | d.f | *F* | *P* |
| Bt | 1 | 434.8 | < 0.001 |  | 1 | 14.098 | < 0.001 |
| β | 1 | 53.8 | < 0.001 |  | 1 | 0.14. | 0.7 |
| Bt x β | 1 | 46.6 | < 0.001 |  | 1 | 0.198 | 0.65 |
