# Supplementary material for: β-carotene and Bacillus thuringiensis insecticidal protein differentially modulate feeding behaviour, mortality and physiology of European corn borer (Ostrinia nubilalis)
Source: PLoS One. 2021 Feb 16;16(2):e0246696. doi: 10.1371/journal.pone.0246696 (PMC7886157; doi:10.1371/journal.pone.0246696)
Supplement: S5 Table — (DOCX) [file pone.0246696.s005.docx]

| **S5 Table**. Student’s *t*-tests on the effect of β-carotene in Non-Bt and Bt diets on the performance of early instar larvae | | | | | | | | | | | |
| --- | --- | --- | --- | --- | --- | --- | --- | --- | --- | --- | --- |
|  | Larval development time | | |  | Larval weight | | |  | Pupal weight | | |
| Diets | d.f | *t* | *P* |  | d.f | *t* | *P* |  | d.f | *t* | *P* |
| Non-Bt vs. Non-Bt-β | 99.3 | -1.77 | 0.079 |  | 62 | -0.083 | 0.934 |  | 47.46 | 1.94 | 0.06 |
| Bt vs. Bt-β | 2.37 | -3.67 | 0.05 |  | 3.38 | 1.13 | 0.331 |  |  |  |  |
