# Supplementary material for: β-carotene and Bacillus thuringiensis insecticidal protein differentially modulate feeding behaviour, mortality and physiology of European corn borer (Ostrinia nubilalis)
Source: PLoS One. 2021 Feb 16;16(2):e0246696. doi: 10.1371/journal.pone.0246696 (PMC7886157; doi:10.1371/journal.pone.0246696)
Supplement: S6 Table — (DOCX) [file pone.0246696.s006.docx]

| **S6 Table**. Three-way ANOVA on the effects of day of quantification and the addition of Bt insecticidal toxin and β-carotene into diets on *O. nubilalis* hormone titre | | | | | | | |
| --- | --- | --- | --- | --- | --- | --- | --- |
|  | 20-Hydroxyecdysone | | | | Juvenile Hormone II | | |
| Variable | d.f | *F* | *P* |  | d.f | *F* | *P* |
| Day of quantification (Day) | 1 | 4.9 | 0.032 |  | 1 | 9.71 | 0.0008 |
| Bt | 1 | 3.87 | 0.05 |  | 1 | 14.89 | < 0.001 |
| β | 1 | 4.77 | 0.035 |  | 1 | 9.78 | 0.046 |
| Bt x β | 1 | 3.92 | 0.05 |  | 1 | 3.05 | 0.08 |
| Bt x Day | 1 | 1.62 | 0.209 |  | 1 | 11.69 | 0.0015 |
| β x Day | 1 | 2.25 | 0.141 |  | 1 | 3.07 | 0.08 |
| Bt x β x Day | 1 | 0.8 | 0.376 |  | 1 | 4.9 | 0.032 |
