# Supplementary material for: β-carotene and Bacillus thuringiensis insecticidal protein differentially modulate feeding behaviour, mortality and physiology of European corn borer (Ostrinia nubilalis)
Source: PLoS One. 2021 Feb 16;16(2):e0246696. doi: 10.1371/journal.pone.0246696 (PMC7886157; doi:10.1371/journal.pone.0246696)
Supplement: S7 Table — (DOCX) [file pone.0246696.s007.docx]

| **S7 Table**. Two-way ANOVA on the effects of Bt insecticidal toxin and β-carotene on *O. nubilalis* hormone titre within days of quantification | | | | | | | | | | | | | | | |
| --- | --- | --- | --- | --- | --- | --- | --- | --- | --- | --- | --- | --- | --- | --- | --- |
|  | 20-Hydroxyecdysone | | | | | | |  | Juvenile Hormone II | | | | | | |
|  | Day 1 | | |  | Day 3 | | |  | Day 1 | | |  | | Day 3 | |
| Variable | d.f | *F* | *P* |  | d.f | *F* | *P* |  | d.f | *F* | *P* |  | d.f | *F* | *P* |
| Bt | 1 | 2.72 | 0.115 |  | 1 | 1.53 | 0.229 |  | 1 | 26.78 | <0.001 |  | 1 | 16.72 | < 0.001 |
| β | 1 | 0.12 | 0.732 |  | 1 | 43.5 | < 0.001 |  | 1 | 2.05 | 0.16 |  | 1 | 3.85 | 0.06 |
| Bt x β | 1 | 2.13 | 0.159 |  | 1 | 3.77 | 0.06 |  | 1 | 4.84 | 0.04 |  | 1 | 4.17 | 0.06 |
