# Supplementary material for: β-carotene and Bacillus thuringiensis insecticidal protein differentially modulate feeding behaviour, mortality and physiology of European corn borer (Ostrinia nubilalis)
Source: PLoS One. 2021 Feb 16;16(2):e0246696. doi: 10.1371/journal.pone.0246696 (PMC7886157; doi:10.1371/journal.pone.0246696)
Supplement: S8 Table — (DOCX) [file pone.0246696.s008.docx]

| **S8 Table**. Student’s *t*-tests on the effect of β-carotene in Non-Bt and Bt diets on the hormone titre of fifth instar larvae between days of quantification | | | | | | | | |
| --- | --- | --- | --- | --- | --- | --- | --- | --- |
|  | 20Hydroxyecdysone | | |  |  | Juvenile Hormone II | | |
| Diet | d.f | *t* | *P* |  |  | d.f | *t* | *P* |
| Non-Bt | 5.38 | -4.44 | 0.008 |  |  | 5.8 | 3.04 | 0.02 |
| Non-Bt-β | 6.23 | -1.56 | 0.16 |  |  | 5.04 | 2.93 | 0.03 |
| Bt | 7.98 | 1.46 | 0.43 |  |  | 5 | 2.57 | 0.049 |
| Bt-β | 7.98 | 1.46 | 0.18 |  |  | 6.76 | -1.41 | 0.2 |
