# Supplementary material for: β-carotene and Bacillus thuringiensis insecticidal protein differentially modulate feeding behaviour, mortality and physiology of European corn borer (Ostrinia nubilalis)
Source: PLoS One. 2021 Feb 16;16(2):e0246696. doi: 10.1371/journal.pone.0246696 (PMC7886157; doi:10.1371/journal.pone.0246696)
Supplement: S9 Table — (DOCX) [file pone.0246696.s009.docx]

| **S9 Table**. Kruskal-Wallis *H*-test of the effect of diet on fifht instar larval weight at after 1 and 3 days of feeding | | | | | | | |
| --- | --- | --- | --- | --- | --- | --- | --- |
|  | Day 1 | | |  | Day 3 | | |
| Variable | d.f | *H* | *P* |  | d.f | *H* | *P* |
| Diet | 3 | 59.69 | < 0.001 |  | 3 | 85.02 | < 0.001 |
