# Supplementary material for: β-carotene and Bacillus thuringiensis insecticidal protein differentially modulate feeding behaviour, mortality and physiology of European corn borer (Ostrinia nubilalis)
Source: PLoS One. 2021 Feb 16;16(2):e0246696. doi: 10.1371/journal.pone.0246696 (PMC7886157; doi:10.1371/journal.pone.0246696)
Supplement: S10 Table — (DOCX) [file pone.0246696.s010.docx]

| **S10 Table**. Mann-Whitney *U*-tests of the effect of diet on larval weight compared between days 1 and 3 | | |
| --- | --- | --- |
| Diet | *U* | *P* |
| Non-Bt | 570 | < 0.001 |
| Non-Bt-β | 560 | < 0.001 |
| Bt | 455 | 0.007 |
| Bt-β | 335 | 0.184 |
